# Genome-wide transcriptional analyses of *Clarireedia jacksonii* isolates associated with multi-drug resistance

**DOI:** 10.3389/fmicb.2023.1266045

**Published:** 2023-09-29

**Authors:** Zhang Huangwei, Jin Peiyuan, Kong Yixuan, Yang Zhimin, Zhou Yuxin, Jung Geunhwa, Hu Jian

**Affiliations:** ^1^College of Agro-Grassland Science, Nanjing Agricultural University, Nanjing, China; ^2^Institute of Botany, Jiangsu Province and Chinese Academy of Sciences, Nanjing, China; ^3^Stockbridge School of Agriculture, University of Massachusetts, Amherst, MA, United States

**Keywords:** RNA-seq, dollar spot, multidrug resistance, *Clarireedia* spp., fungicide resistance

## Abstract

Emerging multidrug resistance (MDR) in *Clarireedia* spp. is a huge challenge to the management of dollar spot (DS) disease on turfgrass. Insight into the molecular basis of resistance mechanisms may help identify key molecular targets for developing novel effective chemicals. Previously, a MDR isolate (LT586) of *C. jacksonii* with significantly reduced sensitivities to propiconazole, boscalid, and iprodione, and a fungicide-sensitive isolate (LT15) of the same species were isolated from creeping bentgrass (*Agrostis stolonifera* L.). The present study aimed to further explore the molecular mechanisms of resistance by using genome-wide transcriptional analyses of the two isolates. A total of 619 and 475 differentially expressed genes (DEGs) were significantly down and upregulated in the MDR isolate LT586, compared with the sensitive isolate LT15 without fungicide treatment. Three hundreds and six and 153 DEGs showed significantly lower and higher expression in the MDR isolate LT586 than those in the sensitive isolate LT15, which were commonly induced by the three fungicides. Most of the 153 upregulated DEGs were xenobiotic detoxification-related genes and genes with transcriptional functions. Fifty and 17 upregulated DEGs were also commonly observed in HRI11 (a MDR isolate of the *C. jacksonii*) compared with the HRS10 (a fungicide-sensitive isolate of same species) from a previous study without and with the treatment of propiconazole, respectively. The reliability of RNA-seq data was further verified by qRT-PCR method using a few select potentially MDR-related genes. Results of this study indicated that there were multiple uncharacterized genes, possibly responsible for MDR phenotypes in *Clarireedia* spp., which may have important implications in understanding the molecular mechanisms underlying MDR resistance.

## 1. Introduction

Dollar spot (DS) is one of the most economically important diseases of turfgrass worldwide (Salgado-Salazar et al., 2018). Multiple species in the genus *Clarireedia* (formerly *Sclerotinia homoeocarpa*) have been reported to cause DS, including *C. homoeocarpa*, *C. bennettii*, *C. jacksonii*, *C. monteithian*, *C. paspali*, and *C. hainanense* (Salgado-Salazar et al., 2018; Hu et al., 2019a,b; Zhang et al., 2022). Management of DS requires repeated inputs of fungicides with different chemical groups. Benzimidazole carbamates (MBCs), dicarboximides (DCFs), 14α-demethylase inhibitors (DMIs), and succinate dehydrogenase inhibitors (SDHIs) are among the most frequently used fungicide classes for DS control in the last few decades (Popko et al., 2012; Stephens and Kaminski, 2019). Due to widespread, repeated uses of fungicidal compounds as well as many other factors such as management location, environmental conditions, genetic regulation, and turfgrass species (Koch et al., 2009; Latin, 2011; Stephens and Kaminski, 2019), resistance to these chemical groups in *Clarireedia* spp. have been reported in many regions around the world (Miller et al., 2002; Jo et al., 2006; Koch et al., 2009; Lee et al., 2017). Moreover, multiple fungicide resistant (MFR) or multi-drug resistant (MDR) populations have emerged in the United States of America, Japan, as well as China, recently (Sang et al., 2015, 2018; Zhang et al., 2021), which makes DS disease increasingly difficult to control.

In the genus *Clarireedia*, resistance mechanisms to different chemical groups of fungicides have been systemically revealed in *C. jacksonii*. Hulvey et al. (2012) found that overexpression of two genes, *ShCYP51B* and *ShatrD* in the DS pathogen *C. jacksonii*, was responsible for reduced DMI fungicide sensitivity. Sang et al. (2016) found that nonsynonymous polymorphisms in codon 366 (isoleucine to asparagine) in histidine kinase gene (*Shos1*) and overexpression of *ShPDR1* led to DCFs resistance in *C. jacksonii* isolates. Hu et al. (2018) found that nonsynonymous polymorphisms at codon 198 and 200 in *β-tubulin* were responsible for high and medium resistance to MBC fungicide thiophanate-methyl, respectively. The H267Y mutation in *SdhB* was determined to be responsible for resistance to boscalid and penthiopyrad in *C. jacksonii* isolates from Japan, and the G91R mutation in *SdhC* was confirmed to be a direct factor conferring resistance to boscalid, fluxapyroxad, isofetamid, and penthiopyrad in *C. jacksonii* isolates from the United States (Popko et al., 2018).

There are two different types of resistance to multiple fungicides: MFR and MDR. Generally, MFR is often caused by accumulation of different mutations at the corresponding fungicide target genes (e.g., *CYP51B*, *β-tubulin*, *os1* and *Sdh* genes in *Clarireedia* spp.), while the mechanisms of MDR in plant pathogenic fungi were often associated with overexpression of single or multiple drug efflux transporters, including ATP-binding cassette (ABC) and major facilitator superfamily (MFS) transporters (Nakaune et al., 2002; Omrane et al., 2015; Sang et al., 2015). In *C. jacksonii*, Sang et al. (2015) demonstrate that MDR is partially attributable to the pleiotropic drug resistance (PDR) transporter gene, *ShPDR1*. Furthermore, they found that a gain-of-function mutation (ATG to ACG at codon 853, M853T) in the fungus-specific transcription factor *ShXDR1* led to constitutive and induced overexpression of cytochrome P450s (CYP450s) and ABC transporters, which are responsible for MDR (Sang et al., 2018). In the previous study, we found that multiple isolates of *C. jacksonii* showed decreased sensitivities to fungicides with different mode of actions (Zhang et al., 2021). Preliminary studies revealed that no consistent mutations were found on the fungicide target genes in the resistant isolates, which suggested that they were MDR rather than MFR isolates. To further characterize if similar mechanisms of MDR responsible for the above-mentioned resistant isolates of *C. jacksonii* found in China, we sequenced *ShXDR1* gene in the sensitive and resistant isolates, but no M853T mutation on *ShXDR1* was found. The mechanisms of MDR in *C. jacksonii* or other species in *Clarireedia* may be complex and need to be thoroughly investigated.

Comparative transcriptomics is a powerful tool to elucidate the molecular mechanisms of fungicide resistance. RNA-seq analyses have been used to uncover the molecular mechanisms of resistance to DMI, SDHI, or MDR in many pathogenic fungi, including *Clarireedia* spp., *Aspergillus fumigates* (Fan et al., 2021), *Fusarium virguliforme* (Sang et al., 2021), and *Cercospora beticola* (Bolton et al., 2016). In this study, the goal is to identify the genes that are differentially expressed between fungicide-sensitive and presumably MDR isolates of *C. jacksonii* under exposures to three different fungicides, propiconazole, boscalid, and iprodione. Comparative transcriptomics was applied to glean insight into the potential variations at transcriptomic level. The results provide additional molecular clues that will aid in elucidating the mechanisms and metabolic pathways related to MDR in *Clarireedia* spp.

## 2. Materials and methods

### 2.1. Fungal isolates

Fungal isolates LT15 and LT586 were recovered from symptomatic leaf blades of creeping bentgrass (*Agrostis stolonifera* L.). Sensitivities of the two isolates to iprodione, propiconazole and boscalid were determined *in vitro* using the respective discriminatory concentrations described in the previous study (Zhang et al., 2021). Agar plugs (5 mm in diameter) were inoculated on PDA plates amended with iprodione (1.0 μg/mL), propiconazole (0.1 μg/mL), and boscalid (10 μg/mL). The non-amended PDA plates served as controls. After 3 days’ incubation, the diameter of colonies was perpendicularly measured twice. The percentage (%) of relative mycelia growth was calculated as the average diameter on fungicide-amended medium divided by the average diameter on non-amended medium and multiplied by 100.

There are several species in the genus of *Clarireedia*. The two isolates tested here were identified to the species level, *C. jacksonii* by sequencing the nuclear ribosomal internal transcribed spacer (ITS) with the universal primer of ITS4 and ITS5 (Supplementary Table S1). ITS has proved useful to differentiate different species in the genus *Clarireedia* in previous studies, and the phylogenetic tree constructed from ITS produced a topology like the combined multi-locus dataset (Salgado-Salazar et al., 2018; Aynardi et al., 2019). Maximum likelihood method was used for constructing phylogenetic tree of select known and published isolates using the Tamura 3-parameter model in MEGA7 and tested with 1,000 bootstrap replicates (Kumar et al., 2016; Hu et al., 2019a,b).

### 2.2. Mycelial preparation and total RNA extraction

The mycelia of the two isolates were prepared for RNA extraction with the method described previously with some modifications (Hulvey et al., 2012). In brief, isolates were inoculated into 25 mL of potato dextrose broth (PDB) with eight agar plugs (5 mm in diameter) and grown for 3 days at 25°C and 150 rpm. Propiconazole (EC, Banner Maxx, Syngenta Crop Protection), iprodione (EC, Rovral, FMC), and boscalid (WG, Cantus, BASF) were added to the tubes with the final concentrations at 0.1, 1, and 10 μg/mL, respectively (Sang et al., 2018; Zhang et al., 2021). The untreated samples were added with same volume of sterile water (Zhang et al., 2021). Tubes were lightly shaken on a benchtop shaker for 1 h. Approximately 100 mg of mycelia for each sample was harvested by vacuum filtration, transferred into a 2-mL screw cap tube, and immediately dropped into liquid nitrogen. Frozen samples were stored at −80°C freezer until further processing. Three biological replicates of each treatment were generated. Total RNA was extracted from each sample (a total of 24 samples) using RNAsimple Total RNA Kit (TIANGEN Biotech, Beijing).

### 2.3. Transcriptome sequencing, assembly, and annotation

Total RNA of each sample was sent to Biomarker Technologies (Beijing, China) for cDNA library preparation and sequencing. RNA concentration and purity were measured using NanoDrop 2000 (Thermo Fisher Scientific, Wilmington, DE, United States). RNA integrity was assessed using the RNA Nano 6000 Assay Kit of the Agilent Bioanalyzer 2100 system (Agilent Technologies, CA, United States). 1 μg RNA per sample was used for constructing cDNA library. Sequencing libraries were generated using NEBNext UltraTM RNA Library Prep Kit for Illumina (New England Biolabs, United States) following manufacturer’s instructions. RNA-seq was performed on an Illumina HiSeq4000 platform (Illumina, CA, United States). After sequencing, reads containing adapter, poly-N or low quality reads were removed through in-house perl scripts^1^ to produce clean data sets. Q20, Q30, GC-content, and sequence duplication level of the clean data were calculated. High quality clean data sets were mapped to the *C. jacksonii* reference genome (Zhang et al., 2023) with Hisat2 software (Kim et al., 2015). Only reads with a perfect match or one mismatch were further analyzed and annotated.

Gene functions were annotated through the following databases: NCBI non-redundant protein sequences (Nr), NCBI non-redundant nucleotide sequences (Nt), Protein family (Pfam), Clusters of Orthologous Groups of proteins (KOG/COG), Swiss-Prot, KEGG Ortholog database (KO), and Gene Ontology (GO).

### 2.4. Functional analysis of differentially expressed genes

Gene expression levels were estimated by fragments per kilobase of transcript per million fragments mapped (FPKM). The formula for FPKM calculation was: FPKM = (cDNA fragments)/mapped fragments (millions) × transcript length (kb). Differential expressed genes (DEGs) were analyzed by using the R package DESeq2 (Love et al., 2014). The value of *p* < 0.05, |log_2_Fold Change| ≥ 1.5 and FPKM > 0.05 were set as the threshold for the identifications of DEGs. Principal component analysis (PCA) and heat map analysis were performed using BMKCloud.^2^ Enrichment analysis of the DEGs was implemented by the R package GOseq based on Wallenius non-central hypergeometric distribution (https://cran.r-project.org/; Young et al., 2021). We used KOBAS software to test the statistical enrichment of the DEGs in KEGG pathways (Bu et al., 2021).

### 2.5. Quantitative real time RT-PCR validation of RNA-seq data

The relative expressions of selected DEGs were assayed with quantitative real-time PCR (qRT-PCR) before and after treatments with propiconazole, iprodione, and boscalid as described above. Total RNA was extracted with RNAsimple Total RNA Kit (TIANGEN Biotech, Beijing), cDNA synthesis from total RNA was conducted with the one-step gDNA removal and cDNA synthesis super mix kit (TransGen Biotech, Beijing). cDNA was diluted to 10-fold, and 1 μL (100 ng) of cDNA which was used for qRT-PCR with HiScript II Q RT SuperMix (Vazyme Biotech, Nanjing). qRT-PCR was conducted on LightCycler 480 II (Roche Life Sciences, Swiss). The actin (*Shact*) gene of *C. jacksonii* was selected as the housekeeping gene, primers for quantifying *Shact* and *ShPDR1* gene were used as reported by Hulvey et al. (2012), and primers for *ShCYP68* gene quantification were used according to Sang et al. (2018). Other primers were designed with Primer5 (https://www.researchgate.net/journal/Biotech-Software-Internet-Report-1527-9162; Supplementary Table S1). The comparative threshold cycle (C*_T_*) method was used for the calculation of relative gene expression. Linear regression analysis of the selected DEGs was performed with FPKM values from RNA-Seq and relative expression values from RT-qPCR.

## 3. Results

### 3.1. Genetic background and fungicide sensitivities of the isolates for RNA-seq

**Figure 1:**
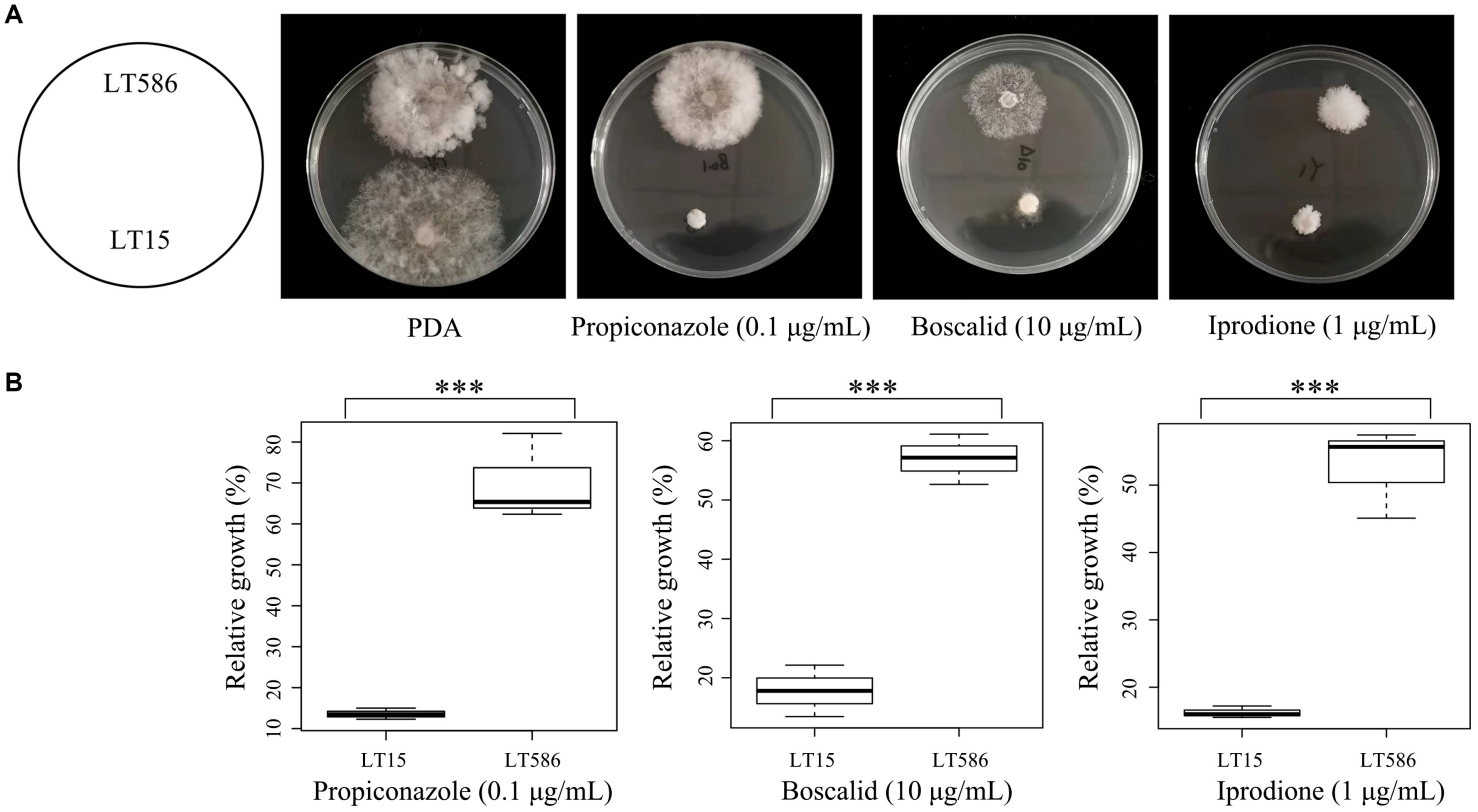
Mycelial growth of the MDR isolate (LT586) and fungicide-sensitive isolate (LT15) of *Clarireedia jacksonii* on non-amended PDA and propiconazole, boscalid, and iprodione amended PDA at 0.1, 10, and 1 μg/mL, respectively. *** means significant difference between the two strains under the fungicide treatment with *p* value < 0.001.

Phylogenetic analysis based on ITS sequences revealed that the two isolates LT15 and LT586 belonged to the same species: *C. jacksonii* (Supplementary Figure S1). *In vitro* sensitivity assays confirmed that the isolate LT586 showed significantly reduced sensitivities to iprodione, propiconazole, and boscalid (Figure 1A) compared with the isolate LT15 (*p* < 0.001) (Figure 1B). No point mutations were found on the fungicide target genes (*ShCYP51B*, *Shβ-tubulin*, *Shos1*, and *ShSdh* genes) in LT586, suggesting that LT586 is a MDR isolate of *C. jacksonii* (Figure 1).

### 3.2. Transcriptomic assembly data

In this study, comparative transcriptomic analyses were conducted to identify the DEGs between the two isolates (LT15 and LT586) without or with the treatments of propiconazole, boscalid, and iprodione (Supplementary Table S2). A total of 39,386,718-59,568,566 clean reads were obtained from 24 libraries. GC content was ranging from 48.75 to 49.23% and the percentage of Q30 base was 93.27% or above (Supplementary Table S2). By iterative alignment, 93.56–95.79% of the clean reads were successfully mapped to *C. jacksonii* reference genome (Zhang et al., 2023). Of the mapped reads, 92.49–94.59% of them was mapped to unique loci, while 0.80–1.26% of the reads was mapped to multiple loci in the reference genome (Supplementary Table S2). The raw RNA sequences were deposited in NCBI SRA Database under BioProject accession number PRJNA1007393.

### 3.3. Differential expression between the two isolates without fungicide treatments

**Figure 2:**
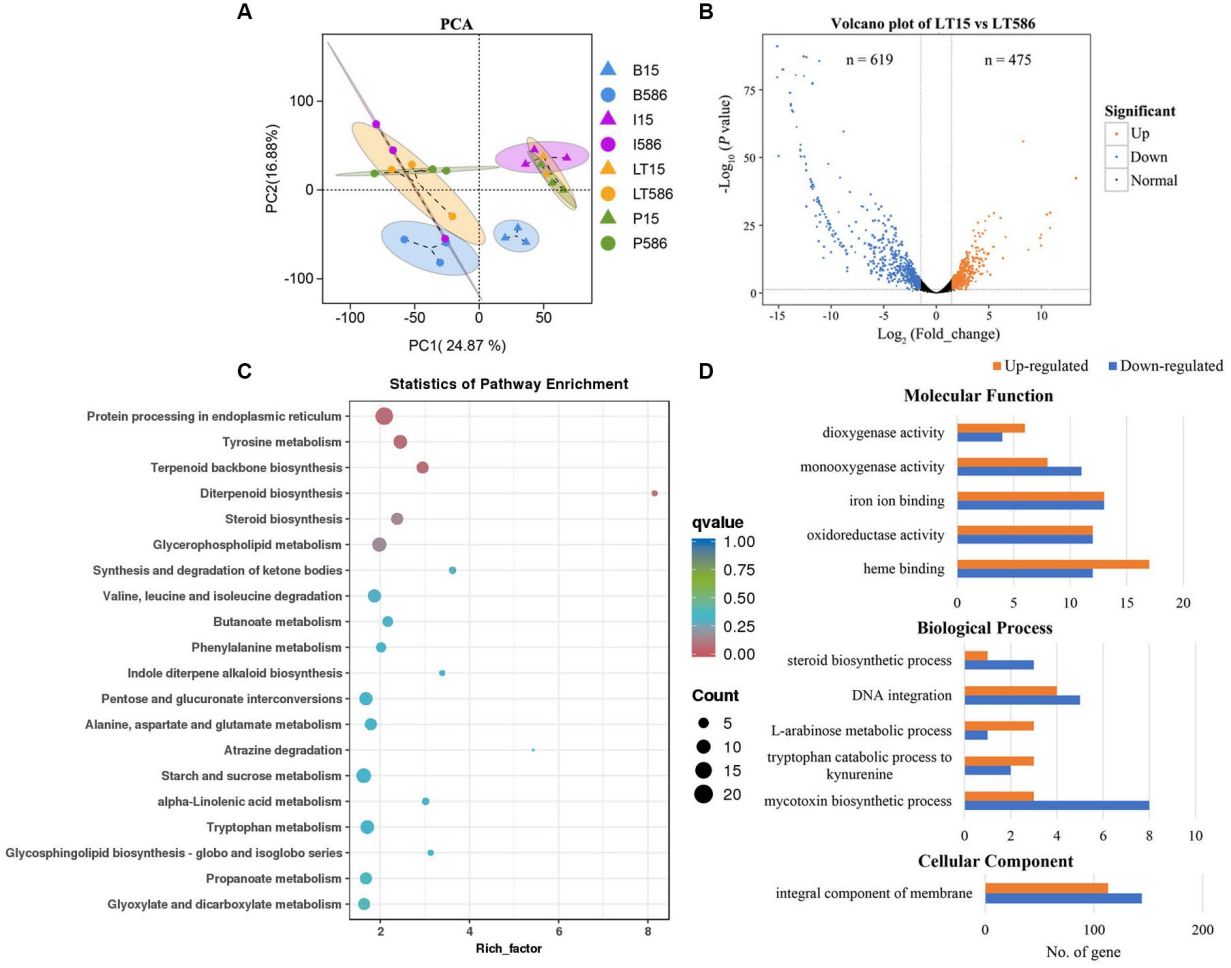
Differential expression analysis between the MDR isolate LT586 and the sensitive isolate LT15 without fungicide treatments. **(A)** Principal component analysis (PCA) of the two isolates with and without the treatments of propiconazole (P15, P586), boscalid (B15, B586), and iprodione (I15, I586), each treatment had three replicates. **(B)** Volcano plot of the DEGs. **(C)** KEGG pathway enrichment analysis of all the DEGs. Rich_factor represents the proportion of genes annotated to a certain pathway in the DEGs compared to all genes annotated, the larger the rich_factor, and the more significance in the enrichment level of the DEGs in this pathway. **(D)** GO functions enrichment analysis of the upregulated and downregulated DEGs.

Principal component analysis revealed that different compartments were clustered apparently, PC1 and PC2 explained 24.87 and 16.88% variation between the two isolates without and with fungicide treatment, respectively (Figure 2A). The DEGs were identified, a total of 619 and 475 DEGs were significantly down- and upregulated in the MDR isolate LT586, compared with the sensitive isolate LT15 without fungicide treatment (Figure 2B).

Clusters of Orthologous Groups of proteins function analysis revealed that all these DEGs were mainly involved in general function prediction (23.29–99%), secondary metabolites biosynthesis, transport, catabolism (12–51%), and energy production and conversion (9.88–42%; Supplementary Figure S2). KEGG pathway of all the DEGs were classified into four groups, which included cellular process, environmental information processing, genetic information processing, and metabolism (Supplementary Figure S3). KEGG pathway analysis revealed that these DEGs were mainly involved in protein processing in endoplasmic reticulum (*n* = 22), terpenoid backbone biosynthesis (*n* = 10), tyrosine metabolism (*n* = 13), diterpenoid biosynthesis (*n* = 3), and steroid biosynthesis (*n* = 10; Figure 2C). GO function analysis showed that the molecular functions of all these DEGs were mainly involved in dioxygenase activity (*n* = 10), monooxygenase activity (*n* = 19), iron ion binding (*n* = 26), oxidoreductase activity (*n* = 24), and heme binding (*n* = 29); the biological processes were mainly involved in steroid biosynthetic process (*n* = 4), DNA integration (*n* = 9), L-arabinose metabolic process (*n* = 4), tryptophan catabolic process to kynurenine (*n* = 5), and mycotoxin biosynthetic process (*n* = 11); the cellular components were mainly involved in integral component of membrane (*n* = 257; Figure 2D).

### 3.4. Differential expression between the two isolates induced by the treatment of three fungicides

**Figure 3:**
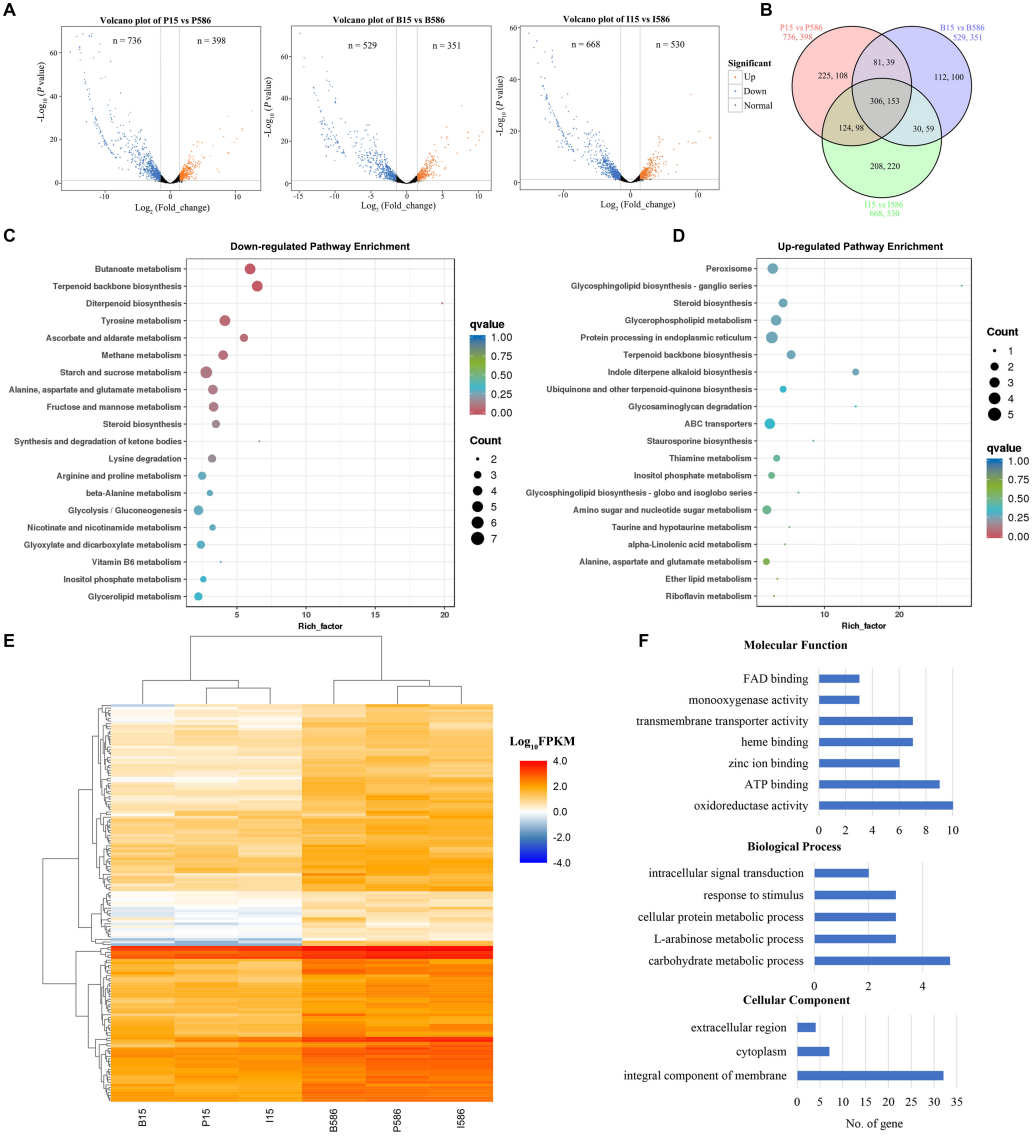
Differentially expressed genes (DEGs) in the MDR isolate LT586 compared with the sensitive isolate LT15 under the treatments of propiconazole (P), boscalid (B), and iprodione (I). **(A)** Volcano plot of the DEGs. **(B)** Venn plot analysis. **(C)** KEGG pathway enrichment analysis of 306 downregulated DEGs commonly induced by the three fungicides. Rich_factor represents the proportion of genes annotated to a certain pathway in the DEGs compared to all genes annotated. The larger the rich_factor, the more significance in the enrichment level of the DEGs in this pathway. **(D)** KEGG pathway enrichment analysis of 153 upregulated DEGs commonly induced by the three fungicides. **(E)** Heat map analysis of the 153 upregulated DEGs commonly induced by the three fungicides. **(F)** GO functions enrichment analysis of the 153 upregulated DEGs commonly induced by the three fungicides.

RNA-seq analyses revealed that 398, 351, and 530 genes were significantly upregulated, while 736, 529, and 668 genes were significantly downregulated in MDR isolate LT586, compared with the sensitive isolate LT15 under the treatment of propiconazole, boscalid, and iprodione, respectively (Figure 3A). A total of 306 and 153 genes showed significantly lower and higher expressions in the MDR isolate LT586 than those in the sensitive isolate LT15, which were commonly induced by the three fungicides (Figure 3B). KEGG pathway enrichment analysis revealed that the 306 downregulated genes were mainly enriched in butanoate metabolism (*n* = 6), terpenoid backbone biosynthesis (*n* = 6), tyrosine metabolism (*n* = 6), and starch and sucrose metabolism (*n* = 7; Figure 3C); the 153 upregulated genes were mainly enriched in peroxisome (*n* = 4), glycerophospholipid metabolism (*n* = 4), protein processing in endoplasmic reticulum (*n* = 5), and ABC transporters (*n* = 4; Figure 3D).

Heatmap analysis showed that the 153 DEGs were clustered apparently in LT15 and LT586, and were significantly higher expressed in the MDR isolate LT586 under the treatments by the three fungicides (Figure 3E). The function of 153 upregulated DEGs further characterized, which were mainly classified into seven molecular function terms, five biological processes, and three cellular components (Figure 3F). The molecular functions of the 153 DEGs were mainly involved in FAD binding, monooxygenase activity, transmembrane transporter activity, heme binding, zinc ion binding, ATP binding, and oxidoreductase activity. The five biological processes were intracellular signal transduction, response to stimulus, cellular protein metabolic process, L-arabinose metabolic process, and carbohydrate metabolic process. The cellular components included extracellular region, cytoplasm, and integral component of membrane (Figure 3F). The specific information of the genes with Pfam function annotations were shown in Supplementary Table S3. Most of them were involved in the functions of xenobiotic detoxification-related genes, which mainly included oxidoreductase activity, transferase activity, ATPase activity, and transmembrane movement of substance.

There were 10 genes involved in oxidoreductase activity (GE10720_g, GE15552_g, GE05437_g, GE03831_g, GE03356_g, GE02832_g, GE09522_g, GE05251_g, GE10755_g, and GE01921_g), which mainly included the functions of alcohol dehydrogenase, zinc-binding dehydrogenase, and NADH: flavin oxidoreductase. Nine genes were involved in ATP binding, including three ABC transporter genes (GE04562_g, GE10243_g, and GE13106_g), one Cdc48 subfamily gene (GE15443_g), one asparagine synthase gene (GE01321_g), one E1-E2 ATPase gene (GE06702_g), two fungal protein kinase genes (GE09508_g, GE16043_g), and one Hsp70 protein encoding gene (GE08240_g). Of the three ABC transporter genes involved in ATP binding (Schoonbeek et al., 2001; Sang et al., 2015), two (GE04562_g = *ShPDR1*, and GE10243_g = *ShatrD*) had been shown to be associated with MDR in *C. jacksonii* (Sang et al., 2018). There were seven genes involved in heme binding, including six CYP450s genes (GE00961_g, GE07314_g, GE05983_g, GE10739_g, GE14553_g, and GE06676_g) and one glycosyl hydrolase family 20 gene (GE05305_g). Three of the six CYP450s genes (GE10739_g = *CYP561*, GE14553_g = *CYP65*, GE00961_g = *CYP68*) were also involved in monooxygenase activity and had been shown to be associated with MDR in *C. jacksonii* (Sang et al., 2018). Seven genes were involved in transmembrane transporter activity, with five genes encoding MFS proteins (GE09014_g, GE09100_g, GE09161_g, GE00853_g, and GE09173_g), one encoding sugar and other transporter protein (GE02436_g), and one encoding POT family protein (GE10772_g). Three of the MFS genes (GE09014_g, GE09100_g, and GE09173_g) were highly homologous with YLL028W (*Tpo1*), YLL028W (*Tpo1*), and YNL065W (*Aqr1*) in yeast (Supplementary Table S3), which had been shown to be associated with fungicide resistance by active efflux of xenobiotics (Kretschmer et al., 2009; Omrane et al., 2015). Three genes were involved in FAD binding (GE05437_g, GE13986_g, and GE13409_g). Eight genes were involved in zinc ion binding (GE05988_g, GE06509_g, GE09053_g, GE09312_g, GE09402_g, GE10720_g, GE12966_g, and GE15454_g), which mainly included the functions of zinc finger domain and fungal specific transcription factor domain.

### 3.5. Comparison with the published data

The transcriptome data from a previously published paper (Sang et al., 2018) were re-analyzed and mapped to the reference genome used in this study (Supplementary Table S2). Three hundred and sixty-nine genes were significantly upregulated in the MDR isolate HRI11, as compared with the sensitive isolate HRS10 under no fungicide-treatment (Supplementary Figure S4A). Fifty of these genes also showed significantly higher expression in LT586 than LT15 from this study (Supplementary Figure S4B). Most of the differentially expressed genes between the two studies were both from the same gene families, such as major facilitator superfamily, CYP450s, and fungal specific transcription factors (Supplementary Figure S4C).

**Figure 4:**
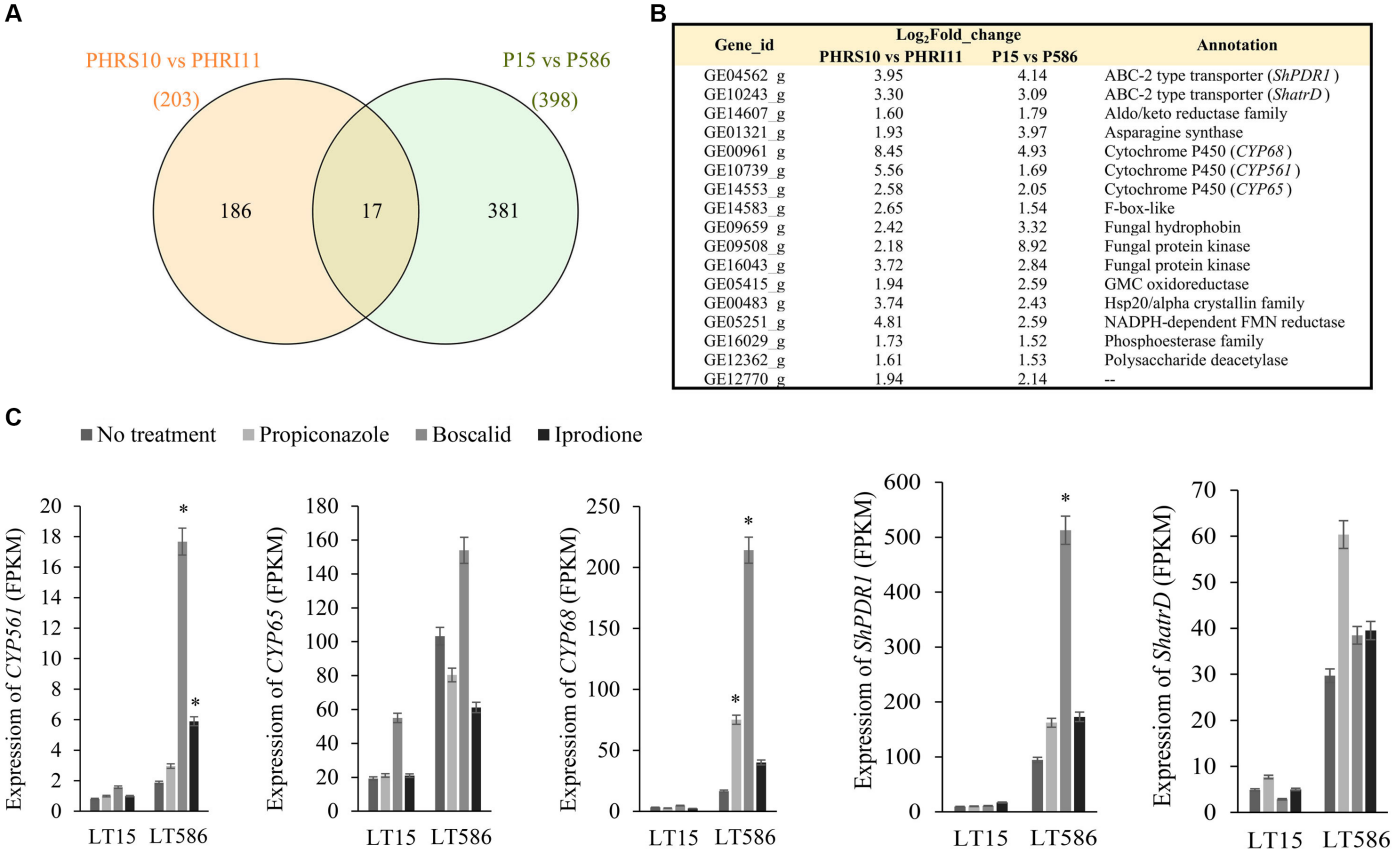
Significantly upregulated genes in HRS10 plus propiconazole vs. HRI11 plus propiconazole and LT15 plus propiconazole vs. LT586 plus propiconazole. **(A)** Venn plot analysis. **(B)** The specific information of 17 commonly upregulated genes through Venn plot analysis. **(C)** Gene expressions of *CYP561*, *CYP65*, *CYP68*, *ShPDR1*, and *ShatrD* in the MDR isolate LT586 and the sensitive isolate LT15 according to RNA-seq data. ^*^means significant difference between the fungicide treatment group and the PDA treatment group in the LT586 isolate value of *p* < 0.05.

In the previously published paper (Sang et al., 2018), *C. jacksonii* isolates were only treated by propiconazole, therefore the transcriptional expression analyses between the two studies were based on the data of propiconazole treatments. A total of 203 (PHRS10 vs. PHRI11) and 398 (PLT15 vs. PLT586) genes were significantly upregulated in the MDR isolates HRI11 and LT586 after treatment of propiconazole (Figure 4A). Seventeen genes showed commonly upregulated overexpression in the two MDR isolates, which included the following five genes (*CYP561*, *CYP65*, *CYP68*, *ShPDR1*, and *ShatrD*), confirmed to be related with MDR function in the MBio paper (Sang et al., 2018; Figure 4B). These genes showed a generally constitutive overexpression and fungicide-induced expression trend in the two MDR isolates, except for *CYP65*, which was only induced expressed under the treatment of boscalid in this study (Figure 4C).

### 3.6. qRT-PCR analysis of key DEGs involved in fungicide resistance

**Figure 5:**
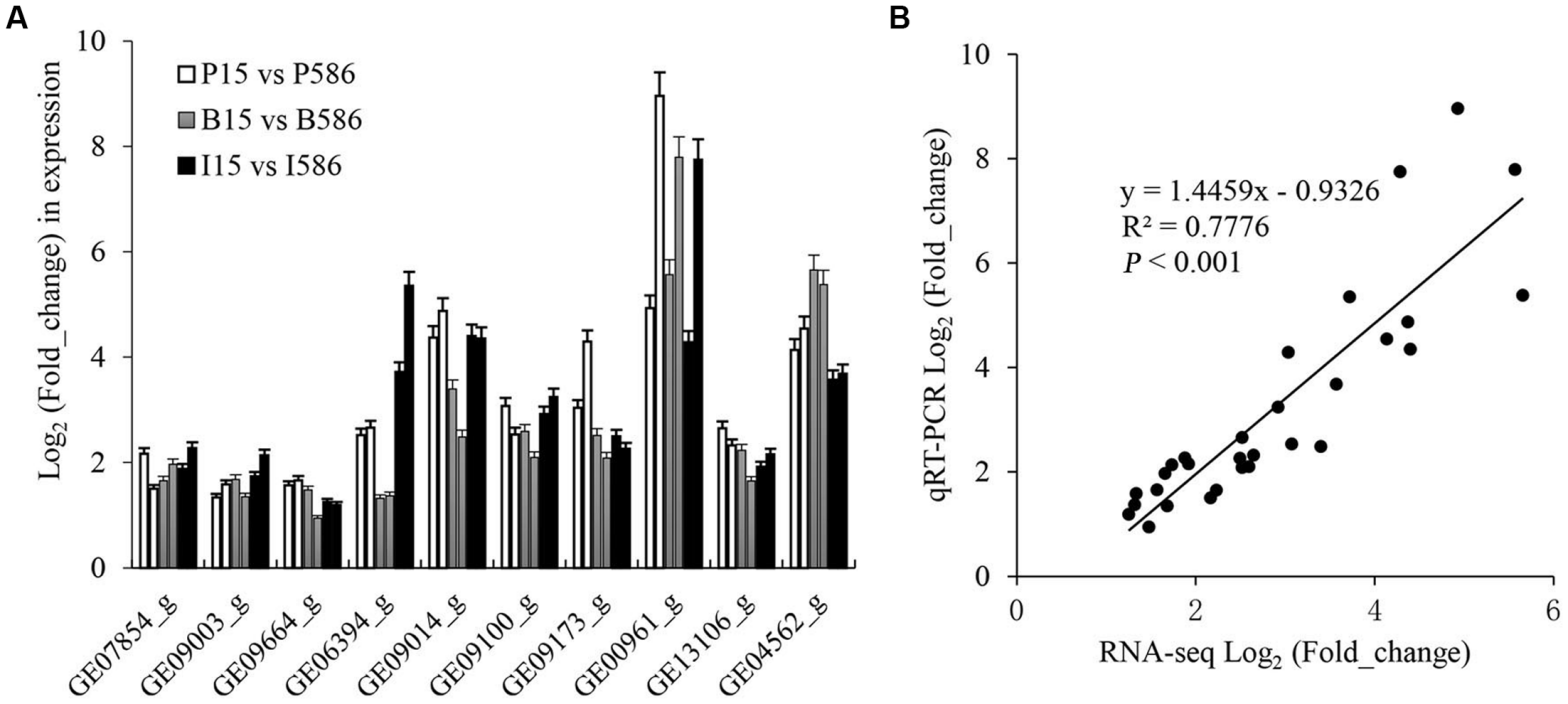
Expression analysis of 10 potentially MDR related genes by qRT-PCR and RNA-seq method. **(A)** Comparison analysis between the two methods, the left and right column with same color refer to RNA-seq and qRT-PCR method, respectively. **(B)** Correlation of the log_2_-transformed relative expression values obtained by qRT-PCR and RNA-seq method.

To validate the expression pattern of some key DEGs involved in fungicide resistance from RNA-Seq analysis, three zinc finger transcript factor genes (GE07854_g, GE09003_g, and GE09664_g), four MFS transporter genes (GE06394_g, GE09014_g, GE09100_g, and GE09173_g), one CYP450 gene (GE00961_g), and two ABC transporter genes (GE13106_g and GE04562_g) were further quantified with qRT-PCR (Figure 5A). The log_2_-transformed relative expression values determined by qRT-PCR and RNA-seq were positively correlated (*R*^2^ = 0.7776, *p* < 0.001; Figure 5B). The result of qRT-PCR analysis was consistent with the RNA-seq data.

## 4. Discussion

*Clarireedia jacksonii* is the most widely distributed DS pathogen species across the world (Viji et al., 2004; Hu et al., 2019a,b). Due to widespread, repeated uses of multiple fungicide classes for DS control, MFR and MDR have become an increasing issue in *C. jacksonii* populations from different regions or countries in recent years (Sang et al., 2016; Stephens and Kaminski, 2019; Zhang et al., 2021). In this study, fungicide sensitivity tests and comparative analyses of fungicide target genes clearly indicated that the *C. jacksonii* isolate LT586 resistant to multiple fungicides was a MDR isolate. At present, MDR in fungal pathogens has become great concerns both in clinical and agricultural fields (Leroux et al., 2012; Sang et al., 2015). Several mechanisms have been reported to cause MDR in pathogenic fungi (Nakaune et al., 2002; Thakur et al., 2008; Kretschmer et al., 2009; Sang et al., 2018). Therefore, we profiled the response of two *C. jacksonii* isolates contrasting in sensitivity to different active ingredients of three fungicide classes: iprodione, propiconazole, and boscalid, to identify the genes involved in MDR. Moreover, we compared the results between this study and a previous study (Sang et al., 2018) to get a better understanding of MDR mechanisms.

This study identified common MDR-related genes, which have also been described in study of Sang et al. (2018), as well as new genes potentially related to MDR. We observed that many genes were constitutively or induced over-expressed by the three fungicides in the MDR isolate LT586 (Figures 2, 3). In general, both constitutive and induced expressions of MDR-related genes can lead to MDR (Sang et al., 2018). Therefore, there were still a substantial number of DEG changes between LT15 and LT586 even in the absence of fungicide treatment. Moreover, the genetic differences also might contribute to the constitutive changes of the DEGs between the two isolates. In this study, both constitutive and induced DEGs were identified between the two isolates, which provided us a better reference to locate the potential MDR genes for further analyses. Among these DEGs identified, some have been previously characterized to be associated with MDR in *C. jacksonii* (Sang et al., 2018), some homologous genes were identified to be responsible for fungicide resistance in other fungi, which have not been studied in *C. jacksonii*. It is proposed that the MDR mechanisms in *C. jacksonii* are complicated and need to be further investigated.

When compared with the data from a similar study conducted by Sang et al. (2018), we found same and novel DEGs between the two studies, which suggested that the MDR mechanisms in *C. jacksonii* might have common as well as contrasting regulatory pathways, depending on the origins of the isolates. There are two main reasons that may contribute to the differences between the two studies. Firstly, the *C. jacksonii* isolates used in the two studies were different in genetic background and fungicide sensitivities. The key genes regulating MDR in these isolates might be selected differently by different ways of fungicide uses or different environment conditions (Koch et al., 2009; Latin, 2011). Secondly, the isolates in the current study were treated by three fungicides, while only propiconazole was used to treat *C. jacksonii* isolates in the study by Sang et al. (2018).

Modifications, over-expression of target genes, and over-expression of efflux transporters in plasma membranes are well known mechanisms of fungicide resistance in filamentous fungi (Sang et al., 2018). Transcriptome analysis in this study revealed that the target gene of DMI, propiconazole (*CYP51*) was significantly upregulated in LT586, while the target genes of SDHI, boscalid (*SdhB*, *SdhC*, and *SdhD*) and dicarboximide, iprodione (*Shos1*) did not show differential expressions between the two isolates (Supplementary Figure S5). Combined with no mutations found on these target genes, indicated that the observed MDR in LT586 was probably caused by the over-expressions of *CYP51* and other genes involved in xenobiotics detoxification systems. A significantly higher level of resistance to propiconazole observed in LT586 further supported partial contribution of *CYP51* to MDR. Further investigations should be oriented to characterize if there exist commonly regulatory factors responsible for over-expressions of *CYP51* as well as the genes in xenobiotics detoxification systems simultaneously.

General xenobiotics detoxification systems work through pathways that can be divided into three phases. Phase I includes metabolizing enzymes such as CYPs, that catalyze xenobiotics, phase II involves conjugating enzymes (e.g., glutathione S-transferase) by adding polar molecules onto compounds (Xu et al., 2005), and in phase III secretion system, ABC transporters or other transmembrane transporters export parent and/or metabolized compounds (Ihunnah et al., 2011). Previous study revealed that the constitutive and induced over-expressions of three phase I metabolizing enzyme genes *CYP561*, *CYP65*, and *CYP68*, two phase III transporter genes *ShPDR1* and *ShatrD* were associated with MDR in *C. jacksonii* (Sang et al., 2018). However, in this study, *CYP65* in the MDR isolate LT586 was only upregulated in response to the treatment of boscalid, but not propiconazole or iprodione. Overexpression of different CYPs displayed various sensitivities to different chemical groups of fungicides, which might be due to the different substrate specificity of CYPs (Moktali et al., 2012). Beside the three above CYPs, three other CYPs were significantly upregulated in the MDR isolate LT586. Further studies are needed to characterize the full functional roles of these differentially expressed CYPs found in this study, which may gain a better understanding of the associations between these different CYPs and MDR. In this study, one phase II glutathione S-transferase (GE09048_g) showed constitutive and induced overexpression in the MDR isolate LT586, this gene was only constitutively overexpressed in the MDR isolate HRS11 in the previous study (Sang et al., 2018). The different expression patterns in different MDR isolates are expected and suggest the role of the gene in fungicide resistance needs to be further investigated.

Notably, the family members including ABC transporters, MFS transporters, zinc-cluster transcription factors, and CYP450s were significantly enriched in these potential multidrug resistant DEGs (Supplementary Table S3). Regarding MFS transporters, they are generally less understood compared with ABC transporters. MFS transporter genes such as GE09014_g, GE09100_g, and GE09173_g in this study (Supplementary Table S3) were highly homologous with YLL028W (*Tpo1*), YLL028W (*Tpo1*), and YNL065W (*Aqr1*) in yeast. *Tpo1* was demonstrated to confer resistance to benzoic acid in *Saccharomyces cerevisiae*, and under the controls of the Gcn4 and Stp1 transcription factors involved in the response to amino acid availability (Godinho et al., 2017). However, the sequences of these three genes are consistent in MDR isolate LT586 and fungicide sensitive isolate LT15 without point mutations. Therefore, the roles of these MFS genes in fungicide resistance and the interactive mechanisms between MFS genes and other transcription factors need to be further studied.

In addition, it has been not well characterized how fungi transcriptionally regulate and coordinate MDR related genes. Transcription factors have been confirmed to play important roles in drug resistance in multiple pathogenic fungi and they generally interacted with downstream target genes or other transcription factors (Nakaune and Nakano, 2007; Thakur et al., 2008; Wang et al., 2018). To date, only the transcription factor ShXDR1 has been demonstrated to regulate downstream MDR related genes in *C. jacksonii* (Sang et al., 2018). Our results indicated that essential and specific roles for other fungal transcription factors in regulating MDR of *Clarireedia* spp. should be investigated (Supplementary Table S3). Further genetic transformation and protein interactive analyses will help better understand if other transcription factors are involved in MDR, and how they regulate MDR in *Clarireedia* spp.

## 5. Conclusion

In this study, we utilized comparative transcriptomics to glean insight into the potential MDR-related genes associated with the reduced sensitivities to multiple fungicides (propiconazole, boscalid, and iprodione) in *C. jacksonii* isolates, comparative analyses were also conducted with the datasets from the current and a previous study (Sang et al., 2018). Common as well as novel DEGs were found to be associated with MDR in this study. The molecular functions of these DEGs were mainly involved in xenobiotic detoxification-related systems and transcriptional regulations. The results obtained from this study will provide valuable information for further identifying the molecular mechanisms of MDR. The study also strongly indicated that the MDR mechanisms in *C. jacksonii* are complicated, which need to be thoroughly characterized through functional studies in the future.

## Data availability statement

The datasets presented in this study can be found in online repositories. The raw RNA sequences were deposited in NCBI SRA Database under BioProject accession number PRJNA1007393.

## Author contributions

ZH: Conceptualization, Data curation, Formal Analysis, Visualization, Writing – original draft. JP: Data curation, Software, Validation, Writing – review & editing. KY: Data curation, Writing – review & editing. YZ: Conceptualization, Supervision, Writing – review & editing. ZY: Conceptualization, Supervision, Writing – review & editing. JG: Supervision, Writing – review & editing. HJ: Writing – review & editing, Conceptualization, Funding acquisition.

## Footnotes

^1^ https://www.perl.org/

^2^ www.biocloud.net
