# Supplementary material for: Genome-wide transcriptional analyses of Clarireedia jacksonii isolates associated with multi-drug resistance
Source: Front Microbiol. 2023 Sep 29;14:1266045. doi: 10.3389/fmicb.2023.1266045 (PMC10570728; doi:10.3389/fmicb.2023.1266045)
Supplement: Supplementary file 4 [file Data_Sheet_1.docx]

Supplementary Figure


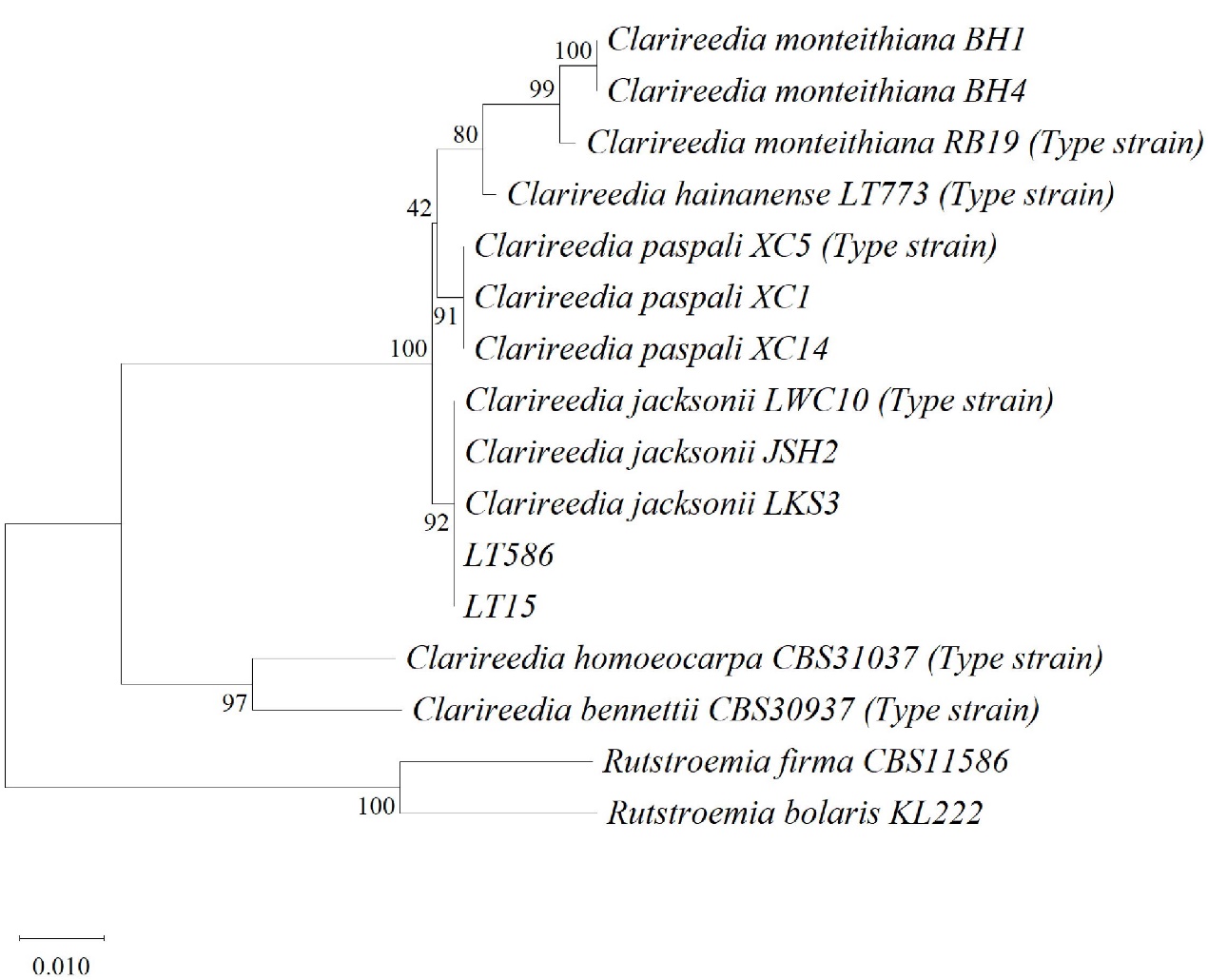


**Fig S1.** Phylogenetic tree from maximum likelihood analysis of the internal transcribed spacer (ITS) sequences. Number at each branch indicates the percentage of occurrences of that branch in 1000 bootstrap replications.


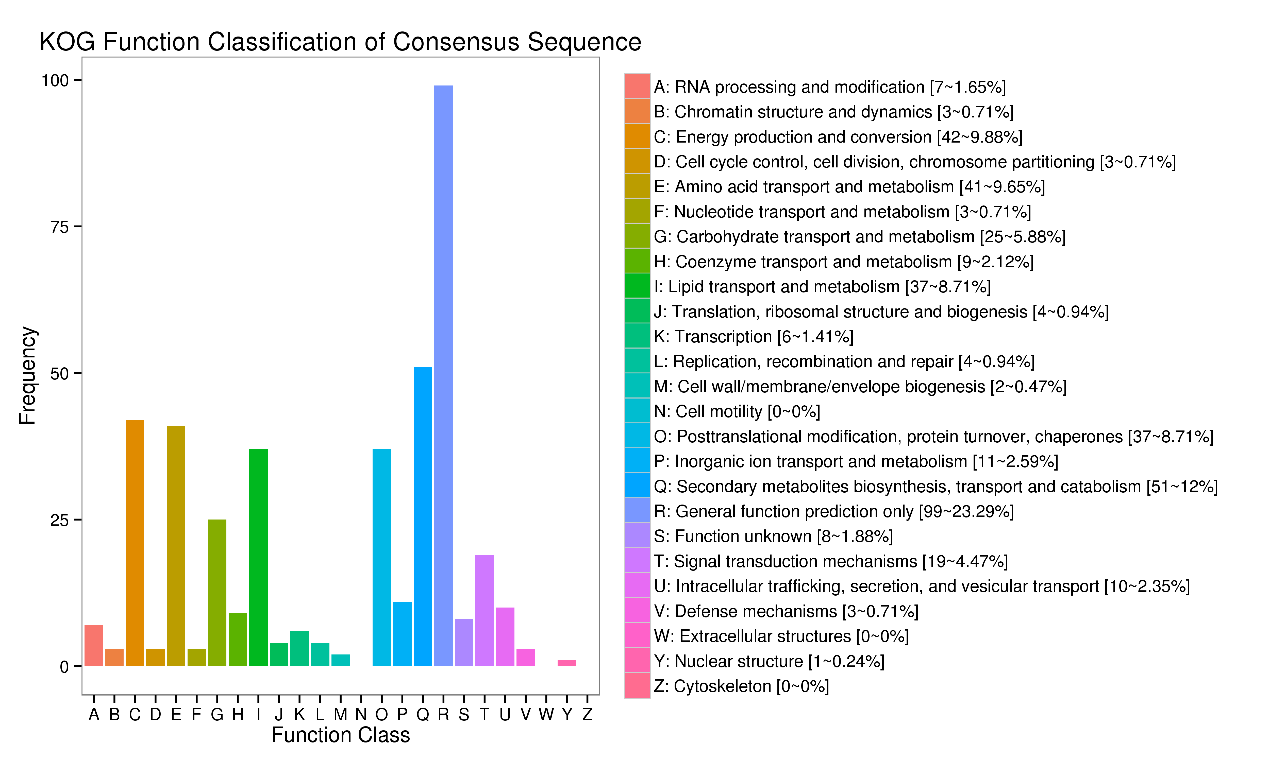


**Fig S2.** KOG function analysis of the differentially expressed genes (DEGs) in the MDR isolate LT586 compared with the sensitive isolate LT15 without fungicide treatment.


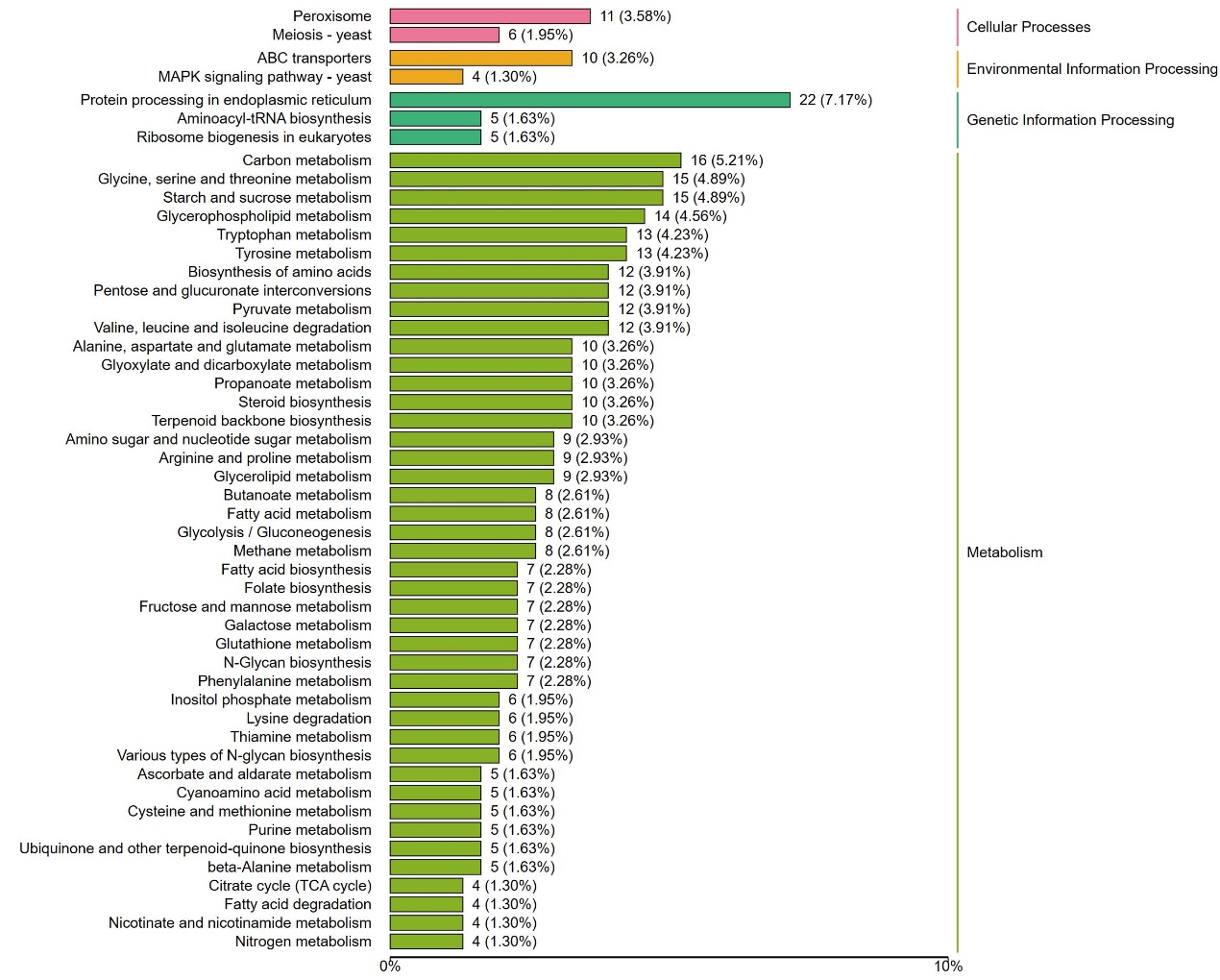


**Fig S3.** KEGG pathway classification of the differentially expressed genes (DEGs) in the MDR isolate LT586 compared with the sensitive isolate LT15 without fungicide treatment.


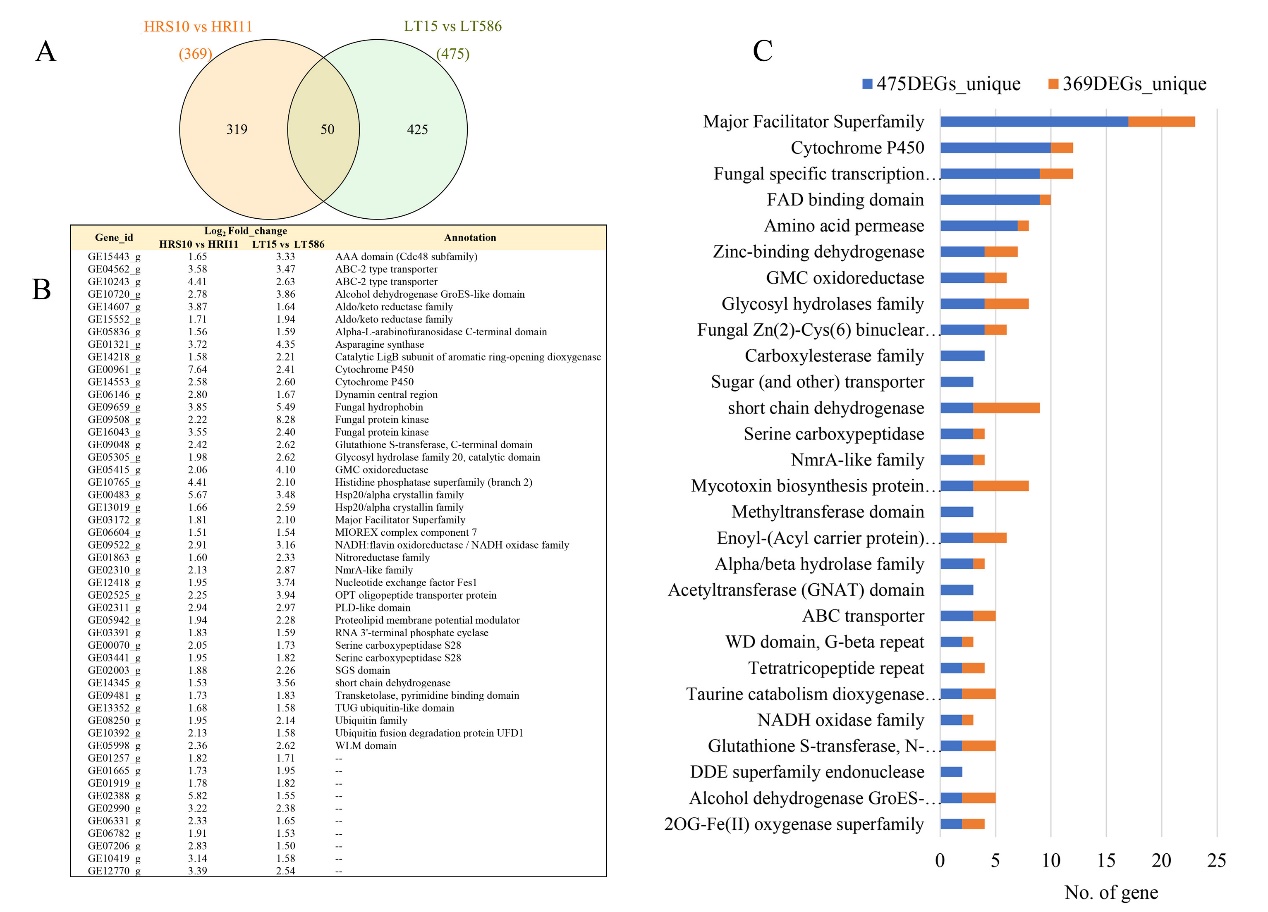


**Fig S4.** Significantly upregulated genes in HRS10 versus HRI11 and LT15 versus LT586 without fungicide treatment. (A), Venn plot analysis. (B), the specific information of 50 commonly upregulated genes through Venn plot analysis. (C), gene family annotations of the significantly upregulated DEGs.


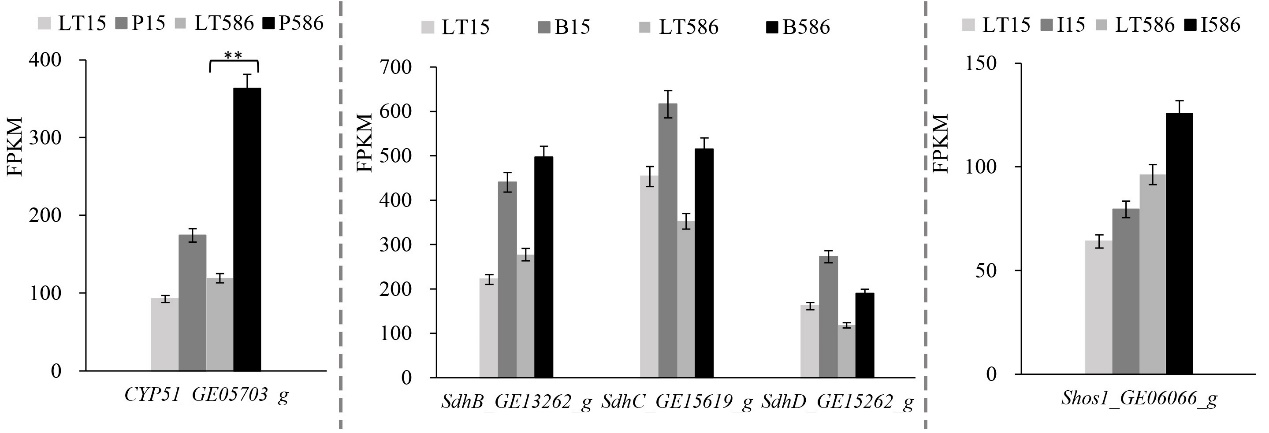


**Fig S5.** Relative expressions of the target genes of propiconazole (*CYP51*), boscalid (*SdhB* *SdhC* and *SdhD*) and iprodione (*Shos1*) in the MDR isolate LT586 and the sensitive isolate LT15 without and with fungicide treatment.
